# Strand-specific transcriptome profiling with directly labeled RNA on genomic tiling microarrays

**DOI:** 10.1186/1471-2199-12-3

**Published:** 2011-01-14

**Authors:** Wen-Han Yu, Hedda Høvik, Ingar Olsen, Tsute Chen

**Affiliations:** 1 Department of Molecular Genetics, The Forsyth Institute, Cambridge, MA, USA; 2 Bioinformatics Graduate Program, Boston University, Boston, MA, USA; 3 Department of Oral Biology, Faculty of Dentistry, University of Oslo, Oslo, Norway

## Abstract

**Background:**

With lower manufacturing cost, high spot density, and flexible probe design, genomic tiling microarrays are ideal for comprehensive transcriptome studies. Typically, transcriptome profiling using microarrays involves reverse transcription, which converts RNA to cDNA. The cDNA is then labeled and hybridized to the probes on the arrays, thus the RNA signals are detected indirectly. Reverse transcription is known to generate artifactual cDNA, in particular the synthesis of second-strand cDNA, leading to false discovery of antisense RNA. To address this issue, we have developed an effective method using RNA that is directly labeled, thus by-passing the cDNA generation. This paper describes this method and its application to the mapping of transcriptome profiles.

**Results:**

RNA extracted from laboratory cultures of *Porphyromonas gingivalis *was fluorescently labeled with an alkylation reagent and hybridized directly to probes on genomic tiling microarrays specifically designed for this periodontal pathogen. The generated transcriptome profile was strand-specific and produced signals close to background level in most antisense regions of the genome. In contrast, high levels of signal were detected in the antisense regions when the hybridization was done with cDNA. Five antisense areas were tested with independent strand-specific RT-PCR and none to negligible amplification was detected, indicating that the strong antisense cDNA signals were experimental artifacts.

**Conclusions:**

An efficient method was developed for mapping transcriptome profiles specific to both coding strands of a bacterial genome. This method chemically labels and uses extracted RNA directly in microarray hybridization. The generated transcriptome profile was free of cDNA artifactual signals. In addition, this method requires fewer processing steps and is potentially more sensitive in detecting small amount of RNA compared to conventional end-labeling methods due to the incorporation of more fluorescent molecules per RNA fragment.

## Background

The oligonucleotide microarray has become a powerful and popular tool for comprehensive transcriptome mapping. Closely tiled probes are suitable for high resolution transcript detection and have been extensively used for discovering novel RNAs including coding, non-coding, antisense, and structural RNAs. However, the conventional methods for transcriptome mapping involve sample processing steps such as reverse transcription, amplification, and ligations which introduce biases and thus may not reveal the true transcriptome profile [1].

In most microarray assays for studying gene expression, RNA is converted to cDNA by reverse transcription and the signal intensities detected from labeled cDNA hybridized to the probes on the microarray are interpreted as the level of transcription. However, reverse transcription has been known for its tendency of generating artifactual cDNA through various mechanisms [1-8]. Furthermore, strand-specific transcription mapping critically relies on first-strand cDNA synthesis and any unintended second-strand synthesis will introduce biases that interfere with both characterization and quantification of the transcripts. To alleviate cDNA artifacts, Perocchi *et al. *[9] added actinomycin D in the reverse transcription which specifically inhibits DNA-dependent DNA synthesis. Comparing the results with or without added actinomycin D, they concluded that approximately half of the antisense transcripts observed in *Saccharomyces cerevisiae *using conventional protocols for first-strand cDNA synthesis (i.e., no actinomycin D added) were experimental artifacts.

To circumvent problems introduced by the reverse transcription, several methods of using RNA directly in microarray hybridization have been developed. For example, Hu *et al. *[10] developed an antibody-based assay for detecting small RNAs. The protocol involved direct RNA hybridization to microarrays followed by RNA-DNA hybrid antibody detection. More recently Dutrow *et al. *[11] described a similar antibody-based assay (HybMap) applied to high-density tiling microarrays for mapping the transcriptome of *Schizosaccharomyces pombe*. Huber *et al. *[12] described an end-labeling technique hybridizing an oligo gold nano-particle probe to the poly(A)-tail of RNAs bound to microarrays. As this method is based on poly(A)-labeling, additional processing is required when applied to prokaryotic RNA, which lacks poly(A)-tails. Enzymatic end labeling methods have also been reported, which involved the use of T4 RNA ligase [13,14] and terminal deoxynucleotidyltransferase [15] to attach biotinylated molecules or labeled nucleotides to the 3' end of the target-RNA. In addition, chemical labeling of nucleic acids using platinum compounds have also been successfully applied to both expression [16,17] and aCGH [18,19] studies. However, platinum labeling only targets guanine in RNA, which may result in uneven labeling densities due to different nucleotide composition of the target molecules.

In this paper we describe a simple, efficient, and sensitive method that chemically labels and uses RNA in microarray hybridization experiments, which can be used for both prokaryotic and eukaryotic transcriptome profiling. The chemical labeling covalently alkylates the RNA on guanine, adenine, and cytosine residues [20] and is thus less affected by the sequence composition of RNA. Compared to enzymatic methods, this chemical labeling requires fewer processing steps and is also free of biases associated with enzymatic reactions. More importantly, the direct use of RNA completely eliminates the cDNA artifacts. We have successfully used this method to map the strand-specific transcriptome of the periodontal pathogen *Porphyromonas gingivalis *using high-density genomic tiling microarrays. This report describes the comprehensive procedure with optimized conditions in all key steps such as RNA extraction, labeling, and microarray hybridization. The data obtained with direct RNA labeling was compared to that of a cDNA-based method and showed only background level of signal in most antisense areas, indicating that most antisense RNA molecules detected with the cDNA-based method were experimental artifacts.

## Methods

### Tiling microarray design

The genomic tiling microarray probe set consisting of 385,000 oligonucleotide sequences was dynamically designed from both forward and reverse-complement strands of the target genome *P. gingivalis *strain W83, using a tiling array probe design algorithm developed by Høvik and Chen [21]. The probe set can be downloaded from the Microbial Transcriptome Database http://bioinformatics.forsyth.org/mtd. The probes were printed on high-density microarrays by Roche NimbleGen, Inc. (Madison, WI, USA).

### Bacterial culture preparation

*P. gingivalis *strain W83 was cultured anaerobically on trypticase soy agar (TSA) plates containing sheep blood, hemin, and vitamin K (BAPHK) [22] for 48 hours at 37°C. Upon harvest, a solution containing 2:1 (v:v) ratio of RNAprotect Bacteria Reagent (Qiagen, Valencia, CA, USA) and 1X PBS was poured onto the colonies and incubated anaerobically for 5 min. The cells were mixed and suspended in this solution, pelleted by centrifugation at 5,000 × g, 4°C for 10 min, and then subjected to either RNA or DNA extraction.

### Total RNA extraction

Lysis of bacterial cells was performed according to the protocol provided with the MasterPure RNA Purification Kit (Epicentre, Madison, WI, USA). The lysate was treated with Proteinase K at 65°C for 15 min and placed on ice. To increase the recovery rate of small RNA, 0.1 volume of 5 M NaCl was added to the lysate. Acid-phenol: chloroform (5:1) (v:v) extraction was then done using Phase-Lock Gel Heavy tubes (Eppendorf, Hauppauge, NY, USA). Total RNA in the upper aqueous phase was purified with a solid-phase extraction filter supplied in the mirVana miRNA Isolation Kit (Applied Biosystems/Ambion, Austin, TX, USA) according to the recommended protocol. The filter-trapped RNA was washed and eluted with the provided wash solutions and elution buffer. To completely remove genomic DNA, the RNA extract was treated twice with Turbo DNase (Applied Biosystems/Ambion) at 37°C for 30 min, and purified again with the mirVana miRNA Isolation Kit.

### RNA direct labeling and microarray hybridization

The *Label *IT Cy3 Reagent (Mirus Bio, Madison, WI, USA) was used to directly label total RNA. To optimize the RNA labeling efficiency, 1 μg RNA was mixed with 4 μl Label IT Reagent and incubated in a 100-μl final volume for 4 hours at 37°C. To improve hybridization efficiency, the labeled RNA was fragmented to an average size of 80-100 nucleotides with 0.25 volume of 5X Fragmentation Buffer (Mirus Bio) and incubated at 94°C for 15 min. The fragmented and fluorescently labeled RNA was purified with the mirVana miRNA Isolation Kit before hybridization.

RNA-DNA hybridization was performed on microarray slides covered with a HybriWell chamber (Grace Bio-Labs, Bend, OR, USA). Prehybridization was carried out for 90 min at 42°C in a 400 μl solution containing 343 μl Long Oligo hybridization buffer [94 mM Tris/HCl pH 7.0, 9.4 mM EDTA, 29.15% formamide, 5.83X SSC, 0.12% SDS], 0.5 mg/ml BSA, and 0.1 mg/ml salmon sperm DNA (Applied Biosystems/Ambion). The salmon sperm DNA was denatured at 95°C for 5 min before added to the prehybridization and hybridization solutions. After the prehybridization the HybriWell chamber was removed and the slide washed with nuclease-free water. The slide was then spin-dried at low speed for 2 min, and a new HybriWell chamber was sealed onto the slide. For each microarray 3 μg of labeled RNA was denatured at 65°C for 5 min in a final volume of 300 μl hybridization solution containing 257 μl Long Oligo hybridization buffer, 3 μl Alignment Oligo (NimbleGen), and 0.7 mg/ml salmon sperm DNA. Denatured RNA was then loaded in the chamber and hybridization was carried out at 42°C for 16 hours with 10 rpm rotation in an oven (Labnet, Edison, NJ, USA). The slide was washed according to NimbleGen's protocol (NimbleGen Arrays User's Guide Gene Expression Analysis).

### cDNA labeling and microarray hybridization

Synthesis of cDNA and biotin end-labeling were performed according to NimbleGen's protocol (Prokaryotic Biotin-Label Procedure). First-strand cDNA was synthesized from 10 μg total RNA. The RNA together with 3 μg random hexamer primers (Invitrogen, Carlsbad, CA, USA) in a 12-μl volume was denatured at 70°C for 10 min. The solution was then cooled to 25°C and mixed to a final concentration of 0.5 mM dNTP, 20 mM DTT, 1X first strand buffer and 0.75 U/μl RNaseOUT (Invitrogen) followed by heating to 42°C. A total of 1200 U SuperScript II reverse transcriptase (Invitrogen) was added to a final volume of 54 μl and the mixture incubated overnight at 42°C. The cDNA product was treated with 0.02 U/μl RNase H (Invitrogen) and 0.01 μg/μl RNase A (Epicentre) in a 100-μl volume to eliminate RNA contaminants. The cDNA was then purified with standard procedures for phenol: chloroform extraction using Phase-Lock Gel Light tubes (Eppendorf) followed by ethanol precipitation. Purified cDNA was fragmented to 50-200 nucleotides in size with DNase I (Applied Biosystems/Ambion), and labeled at the 3'end in a 100-μl volume containing 0.5 U/μl Terminal Deoxynucleotidyl Transferase (Promega, Madison, WI, USA) and 0.025 mM Biotin-N6-ddATP (Enzo Life Sciences Inc., Farmingdale, NY, USA). The labeling reaction was carried out for 2 hours at 37°C. The biotin-labeled cDNA was then concentrated using Microcon YM-10 filters (Millipore, Billerica, MA, USA). Microarray hybridization was carried out with the customer service provided by NimbleGen.

### Genomic DNA extraction and hybridization

Genomic DNA for DNA reference microarray hybridization was extracted with the MasterPure DNA Purification Kit (Epicentre) and RNA was removed using the RNase A supplied in the kit. Genomic DNA was fragmented with DNase I (Applied Biosystems/Ambion) to 100-200 nucleotides in size and then labeled either with biotin 3'end-labeling (i.e., cDNA procedure) or with the *Label *IT Reagent (i.e., direct RNA labeling). Conditions for microarray hybridization were the same as described in the previous sections for cDNA or RNA hybridization, respectively.

### Data acquisition and normalization

After washing and drying, the microarray slides were immediately scanned in a GenePix 4000B Scanner (Axon Instruments, Union City, CA, USA) using the provided GenePix Pro 6.1 software. NimbleScan v2.5 software was then used for spot features extraction from the scanned images. Each of the microarray hybridization results hybridized with RNA, cDNA, or genomic DNA, consisted of at least two biological repeats. Two types of normalizations were performed - normalization by DNA reference array and between-array normalization. For DNA reference array normalization, intensities of cDNA and RNA signals were normalized with signals from DNA reference arrays that were hybridized with fragmented genomic DNA labeled in the corresponding way, i.e., biotin end-labeling and chemical labeling for cDNA and RNA, respectively. Both normalizations were done using the Bioconductor R package "tilingArray" [23]. Coding and non-coding regions were determined based on the annotation of the *P. gingivalis *W83 genome available from the National Center for Biotechnology Information (NCBI, http://www.ncbi.nlm.nih.gov). The baseline (i.e., background level) of each resulting intensity profile was calculated and represents the average value of the probe signal intensities from all intergenic regions. Intensities of probe sequences falling within 200 nucleotides to both ends of the intergenic sequences were excluded from the calculation to avoid possible positive signals from either 5'- or 3'end untranslated regions.

### Data availability and deposition

Original and normalized microarray data used in this paper were deposited in the NCBI Gene Expression Omnibus (GEO) database http://www.ncbi.nlm.nih.gov/geo, with accession ID GSE25876. The transcriptome profiles are also available for viewing at the "Microbial Transcriptome Database" website, http://bioinformatics.forsyth.org/mtd.

### Strand-specific RT-PCR using tagged primers

In conventional reverse transcription PCR (RT-PCR), false positive PCR artifacts have been reported in absence of primer used in the reverse transcription step, due to self-priming of RNA or non-specific small DNA/RNA oligonucleotide contaminants that can also be used as primers in RT reactions [6]. To avoid this problem, first-strand cDNA was synthesized with a genome specific primer attached with a tag sequence to the 5'end [24]. The tag sequence was unique and not found in the genome of *P. gingivalis*. The subsequent PCR was then carried out with the tag sequence as one of the paired primers. Sequences of the primers used are listed in Table S1, Additional file 1. As a result, only the cDNA synthesized with the tagged primer can be amplified. A separate set of primers for amplifying the sense strand of one of the housekeeping genes in *P. gingivalis *- *mutB*, was included as positive control in all RT-PCRs. Negative controls were performed without the addition of reverse transcriptase to the RT reactions. For RT reactions, 2 μg RNA and 2 μl of both tagged and *mutB *RT-primers (2 μM) were mixed in a 12-μl volume, denatured by heating at 65°C for 5 min, and chilled on ice. To the RNA/primer solution the following reagents were added to a final concentration of 0.5 mM dNTP, 20 mM DTT, 1X first strand buffer, and 2 U/μl RNaseOUT (Invitrogen). The solution was heated to 50°C and mixed with 200 U of SuperScript III reverse transcriptase (Invitrogen) in a final volume of 20 μl. The mixture was incubated at 50°C for 50 min and the RT was terminated by heat inactivation for 5 min at 85°C. To remove RNA, RNase A (Epicentre) was added to a final concentration of 0.01 μg/μl and incubated for 10 min at 37°C. RT-primers were then removed using the MinElute PCR Purification Kit (Qiagen). PCR amplification was performed in a 20-μl volume containing 1.2 μl of the RT product, 17 μl Platinum Blue PCR SuperMix (Invitrogen), and 1 μl of each forward and reverse primers (10 μM). The thermal cycling conditions were: 2 min at 95°C followed by 25 cycles of 30 sec at 95°C, 30 sec at 55°C, and 20-90 sec (depending on PCR product length) at 72°C.

## Results

### Comparison of transcriptome profiles revealed by labeled RNA and cDNA

**Figure 1:**
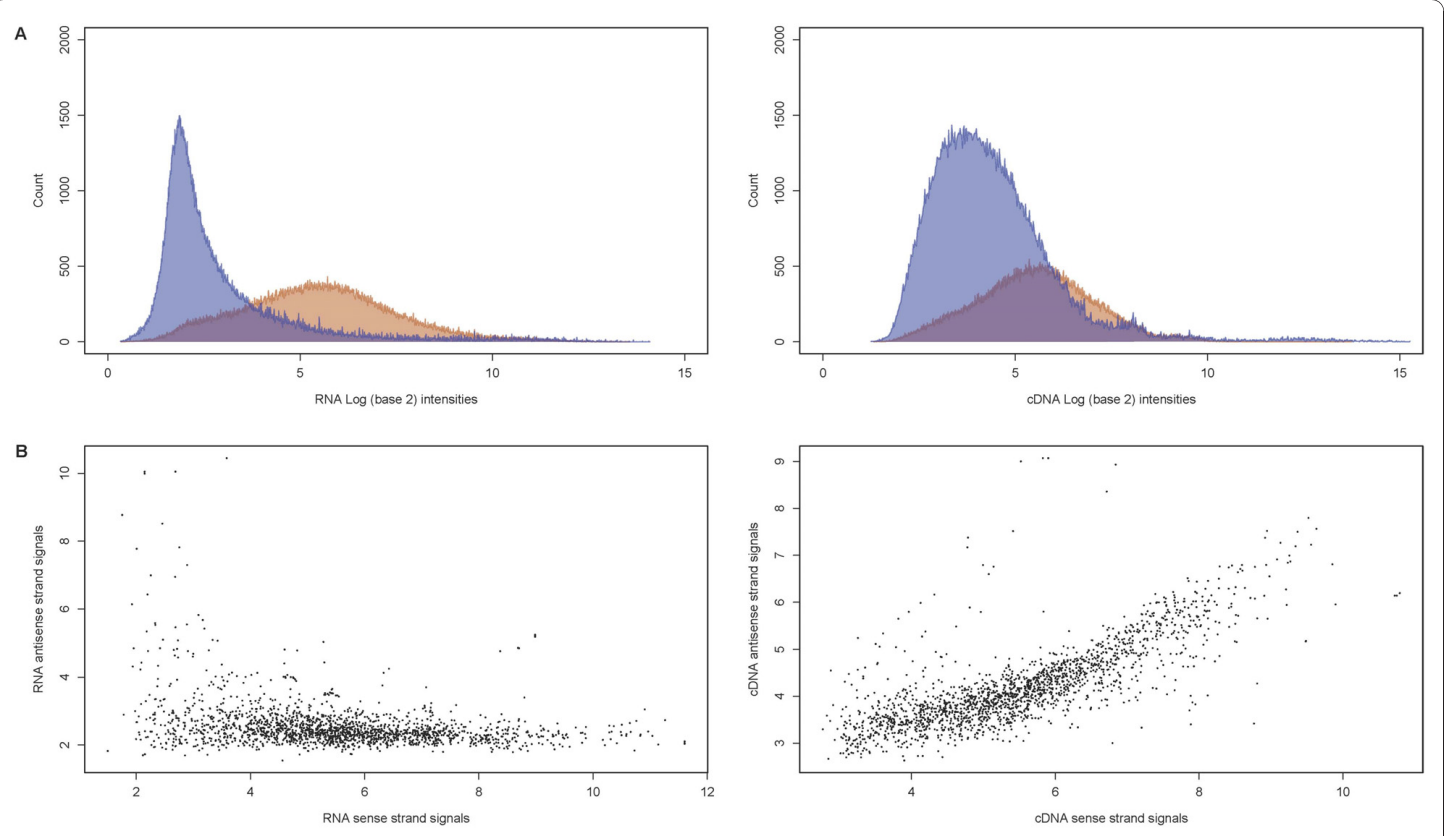
**Comparison of RNA- and cDNA-based hybridization signals**. A: Histograms of log2 probe signal intensities obtained from the RNA-based (left panel) and cDNA-based method (right panel). Probe intensities from coding and non-coding regions highlighted red and blue, respectively. B: Scatter plots of signal intensities between those from the coding (x axis) and corresponding antisense (y axis) regions of the genome. RNA-based signals were plotted in the left panel and cDNA-based in the right.

RNA isolated from *P. gingivalis *cells was directly labeled by alkylation and hybridized with oligonucleotide probes on the microarrays. The derived transcriptome profile (RNA-based profile) was compared to that derived from the biotin end-labeled cDNA (cDNA-based profile). Figure 1A shows the distributions of probe intensities corresponding to coding and non-coding regions after RNA and cDNA microarray hybridizations. The two signal distributions from the RNA-based profile appeared well separated, whereas those of the cDNA-based profile overlapped significantly with an increase of signal intensity for non-coding regions. Figure 1B illustrates the correlation of signal intensities between the coding and corresponding antisense (y axis) regions of the genome. No correlation was observed for the RNA-based profile, while the scatter plot for the cDNA-based profile exhibited positive correlation between coding and corresponding antisense signal intensities. This positive correlation was most likely due to increased antisense signals. Results obtained from the cDNA-based profiles in terms of signal intensities from non-coding and antisense regions suggested the presence of artifactual cDNA signals.

**Figure 2:**
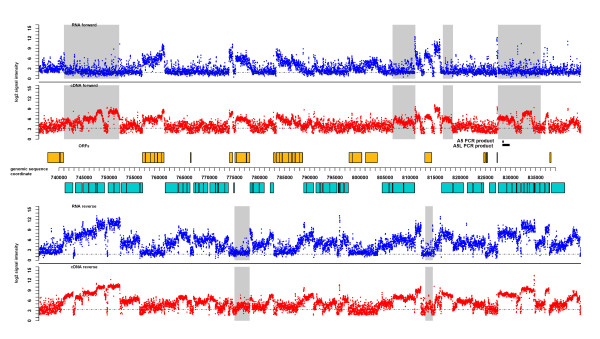
**Comparison of RNA- and cDNA-based transcriptome profiles**. Normalized log2 probe signal intensities (y axis) from a 100-kbp region of *P. gingivalis *genome were plotted on the genomic sequence coordinate (x axis) based on the positions of the probe sequences in the genome. The genomic positions of open reading frames (ORFs) within this region from both forward (orange boxes) and reverse (turquoise boxes) strands are shown in the middle of the figure. The gray dashed line in each of the four profiles represents the baseline. The regions highlighted in gray contain potential artifactual cDNA signals from the antisense strand of the corresponding ORF. The black bars labeled "A5/A5L PCR product" depict the positions and sizes of the expected strand-specific RT-PCR results referred to in Figure 3.

The transcriptome profile was compiled by plotting the normalized hybridization signal intensities on the genomic coordinate based on the probe positions. Forward and reverse-complement probe signals were plotted separately and reflect the genome-wide level of RNA transcribed in the cells. Figure 2 presents a sample region of the transcriptome profile. Both RNA- and cDNA-based profiles displayed similar topology with positive signals corresponding to most ORFs. However, in the antisense strand of most genes, the probe intensities of the cDNA-based profile were significantly higher than those of the RNA-based profile (e.g., regions highlighted grey in Figure 2).

### Determination of sensitivity and optimization of method conditions

In a time-series experiment we observed that the incubation time was linearly proportional to the labeling density of the RNA molecules during the first four hours (Figure S1, Additional file 1). The labeling conditions (described in the Methods section) with an incubation of four hours generated a density of one fluorescent label per 20 nucleotides. Hence, with RNA fragments of 80-100 nucleotides in size, on average 4-5 labels were attached to each RNA molecule.

In our hands, optimal signal-to-noise ratio of the probe intensity distribution was achieved in a hybridization solution with 25% formamide at 42°C. RNase-free BSA was added to the pre-hybridization solution and salmon sperm DNA was included in both pre-hybridization and hybridization solutions to block non-specific binding of RNA molecules to the probes and surface of the microarray slide. We achieved optimal effect when adding 0.7 mg/ml blocking reagent (i.e., salmon sperm DNA) to the hybridization (Figure S2, Additional file 1).

### Validation of RNA signals by strand-specific RT-PCR

**Figure 3:**
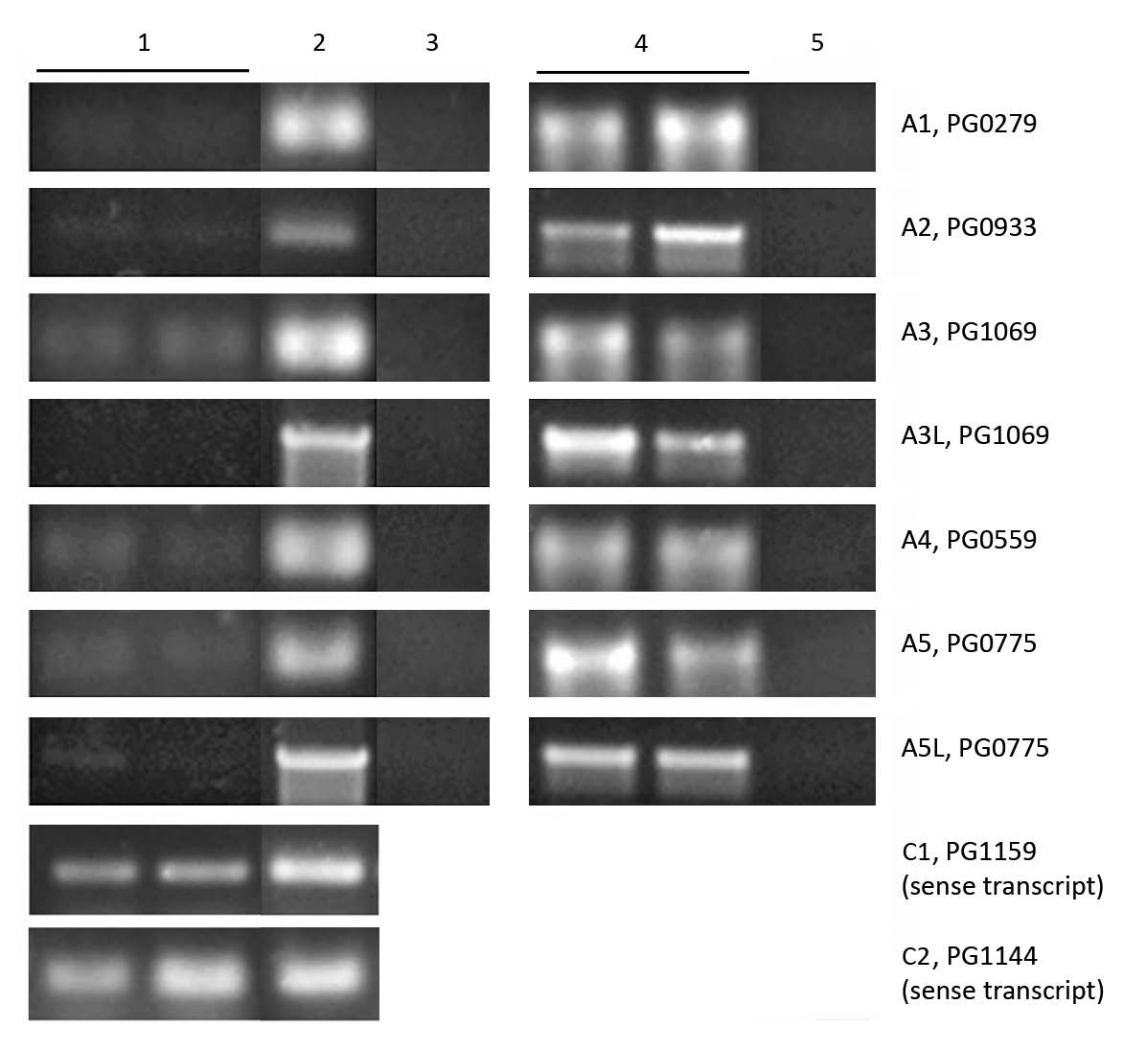
**Strand-specific RT-PCR targeting *P. gingivalis *W83 genes**. Antisense sequences targeted by RT-PCR: PG0279, PG0933, PG1069, PG0559, and PG0775 (marked A1-5, respectively). A3L and A5L were longer PCR products from PG1069 and PG0775. C1, PG1159 and C2, PG1144 were sense sequences targeted by RT-PCR. Columns from left to right: 1, RT-PCR amplicons on RNA duplicates for the targeted antisense and sense sequences; 2, PCR of the targeted antisense and sense sequences on gDNA; 3, RT-PCR of the targeted antisense sequences without reverse transcriptase; 4, RT-PCR amplicons on RNA duplicates targeting the *mutB *gene; 5, RT-PCR of *mutB *without reverse transcriptase.

To verify the transcription signals detected by either the RNA- or cDNA-based method, especially in the antisense regions where cDNA artifactual signals were often reported, strand-specific RT-PCRs using tagged RT-primers were performed. The antisense strand of five highly expressed ORFs was targeted: PG0279, PG0933, PG1069, PG0559, and PG0775 (marked A1-5, respectively). Strong antisense signals were detected in these regions from the cDNA-based profile while only near background levels of signals were found in the corresponding areas of the RNA-based profile (i.e., Figure 2, A5 locus). RT-PCR results for the targeted antisense regions are presented in Figure 3. There was always a positive signal for the housekeeping gene *mutB *validating the RT-PCR conditions and the quality of the RNA samples. However, there was no or weak amplification for each of the targeted antisense regions. The RT-PCR products C1 and C2 were strongly amplified from the coding/sense strand of the ORFs PG1159 and PG1144. Their signal intensities from the cDNA-based profile were close to those of the selected antisense targets. Hence, based on the cDNA signals, the RT-PCR results (A1-5) should be similar to those of C1 and C2 in terms of the intensities of the PCR products. This was not supported by the results. The fact that no or weak signals were detected for the five targeted ORFs indicates that there was either none or only a trace amount of RNA transcribed from these antisense regions. The signal intensities from the same areas of the RNA-based profile were close to background level and thus reflected more accurate levels of RNA in the sample. The faint PCR bands detected by RT-PCR may have been derived from trace amount of RNA present or be caused by artifactual cDNA generated in the reverse transcription through other mechanisms.

## Discussion

We have described a comprehensive experimental procedure with conditions optimized specifically for studying strand-specific transcriptome profiles using genomic tiling microarrays. Our results, including the comparison of RNA- and cDNA-based transcriptome profiles and the detection of transcripts by RT-PCR, confirmed that the use of fluorescently labeled RNA generated a profile without artifactual antisense signals, revealing a better transcriptome profile than the use of cDNA. However, RNA is labile and susceptible to degradation, thus extra cautions are needed to prevent the degradation of RNA and reduction of signals. In addition, strong secondary structures of RNA molecules may reduce hybridization efficiency [25]. To minimize secondary structural effects and to increase the hybridization signal intensity, RNA samples were fragmented to an average size of 80-100 nucleotides. During RNA-DNA hybridization formamide was added to increase both sensitivity and specificity of the hybridization between probe and target RNA [26]. Formamide can help suppress secondary structures of both probes and targets, and improve hybridization by disrupting the hydrogen bonding [27]. Through the same mechanism, a perfectly matched duplex should be less affected by formamide than a duplex with mismatches; hence, formamide potentially improves the ratio of specific to non-specific hybridization. Blocking agents are often used in the hybridization solutions to prevent non-specific binding [28,29]. With the addition of blocking agents to the hybridization solutions, we observed reduced background noise at baseline level and increased sensitivity for detecting true RNA signals.

The fluorescent labeling density directly affects the signal intensity, and thus the overall sensitivity for detecting RNA, especially RNAs transcribed at low levels. The sensitivity of the labeling technique we used (i.e., one fluorescent label per 20 nucleotides) is potentially five times greater than that of single dye end labeling [14] based on RNA fragments averaging 100 nucleotides in size.

The most important advantage of using labeled RNA directly in microarray hybridization for detecting transcription signals is the elimination of cDNA artifacts. The generation of artifactual cDNAs in reverse transcription is due to several possible mechanisms including self-priming or non-specific oligonucleotides priming to the newly generated first-strand cDNA [4,6], template switching [5], primer-independent cDNA synthesis [7], and error-prone transcription of cDNA [8]. A known method to reduce the unintended cDNA generation is to add actinomycin D (ActD) to the RT reaction [9]. ActD inhibits the second-strand cDNA synthesis possibly through the binding of deoxyguanosine residues on cDNA [30]. However, ActD will not prevent other mechanisms causing unintended reverse transcription. As a test we included ActD at 6 μg/ml in the RT reaction. Scatter plots of the signal intensities between the RNA- and cDNA-based profiles show that even with added ActD no significant reduction of overall antisense signals was observed when compared to the RNA-based intensities (panels A and B, Figure S3, Additional file 1). The signals coming from the antisense regions still display positive correlation between those done with or without ActD (panel C, Figure S3, Additional file 1). The concentration of ActD tested may not be sufficient or optimal. However, we observed an inhibitory effect of ActD on the generation of first-strand cDNA at higher concentrations (Figure S4, Additional file 1). Even if increasing the concentration of ActD will inhibit second-strand cDNA generation, it may also compromise the efficiency of first-strand cDNA synthesis. Hence, the advantage of using ActD may be outweighed by the disadvantage.

In addition to the experimental procedure, post-scanning data processing is also important in maximizing the quality of the transcriptome profile [31]. The binding signals detected by hybridization are based on nucleic acid sequence homology and are affected by various factors such as secondary structures and compositions of the probe and target sequences [25,32]. To reduce the effect of these factors, both RNA- and cDNA-based signal intensity data were subjected to two types of normalization. In the normalization with DNA reference arrays, genomic DNA hybridization signals were used to reduce the degree of RNA signal intensity fluctuation by correcting probe sequence composition variations [23]. Background noise estimated from genomic DNA reference signals provides experimental corrections for sequence-specific factors, including different thermodynamic properties corresponding to probe sequence composition [31], bias in labeling efficiency, and the abundance of target sequences. The between-array normalization used in this study was based on the "vsn" algorithm, also available in the R "tilingArray" package [23], and facilitated the comparison of intensity profiles derived from different arrays or experiments.

The final step for compiling a transcriptome profile is the determination of transcription boundaries between expression and non-expression signals. We have developed a dynamic algorithm specifically for this purpose and have used it to annotate the transcriptome profiles obtained in this study. The detailed description of this algorithm has been published elsewhere [33].

## Conclusions

A comprehensive procedure for mapping transcriptome profiles specific to both strands of a genome was developed. Chemically labeled RNA was used directly in the microarray hybridization. Hence, experimental artifacts induced by cDNA synthesis were eliminated and the generated transcriptome profile was free of cDNA artifactual signals. In addition, this method requires fewer processing steps and is potentially more sensitive in detecting low level RNA expression compared to conventional end-labeling methods due to the incorporation of more fluorescent molecules per RNA fragment. The complete RNA-based *P. gingivalis *W83 transcriptome profile is available for viewing at the "Microbial Transcriptome Database" website, http://bioinformatics.forsyth.org/mtd.

## Competing interests

The authors declare that they have no competing interests.

## Authors' contributions

WY and HH performed the experiments in this study. All authors contributed to the study design, data processing, and the writing of the manuscript. All authors read and approved the final manuscript.

## Supplementary Material

Additional file 1 **The additional file includes a table listing the primer sequences used in this work and four figures showing additional results including the efficiency of RNA labeling, the effects of blocking reagent on the background signals, and the effects of actinomycin D added in the reverse transcription reaction**. Click here for file
