# Supplementary material for: Strand-specific transcriptome profiling with directly labeled RNA on genomic tiling microarrays
Source: BMC Mol Biol. 2011 Jan 14;12:3. doi: 10.1186/1471-2199-12-3 (PMC3031212; doi:10.1186/1471-2199-12-3)
Supplement: Additional file 1 — The additional file includes a table listing the primer sequences used in this work and four figures showing additional results including the efficiency of RNA labeling, the effects of blocking reagent on the background signals, and the effects of actinomycin D added in the reverse transcription reaction. [file 1471-2199-12-3-S1.DOC]

| **Label and Locus tag** | **RT/PCR-primers ^1,2^** | **Sequence of primers (5'-3')** | **PCR product  length (bp)** |
| --- | --- | --- | --- |
|  |  |  |  |
| A1, PG0279 | Tagged RT-primer | GGC AGT GCG TGA ATA TGT TGC GGG ACT GAT GAG CCA CCT TA | 171 |
|  | Gene-specific PCR-primer | TCC CGA ATT TGC TTC TCA TC |  |
|  | Tag PCR-primer | GGC AGT GCG TGA ATA TGT TGC |  |
|  |  |  |  |
| A2, PG0933 | Tagged RT-primer | GGC AGT GCG TGA ATA TGT TGC GGA GCC TGT CTA TGA TGT GGA | 178 |
|  | Gene-specific PCR-primer | AGA GCT GAG CGA AGT GGA AT |  |
|  | Tag PCR-primer | GGC AGT GCG TGA ATA TGT TGC |  |
|  |  |  |  |
| A3, PG1069 | Tagged RT-primer | GCC AAG GTA ACT GAG AAT GAC TCT GCA CGA AGA ATG ATG G | 170 |
|  | Gene-specific PCR-primer | GCC TCA CAT TCT CGA AGC TC |  |
|  | Tag PCR-primer | GCC AAG GTA ACT GAG AAT GA |  |
|  |  |  |  |
| A3L, PG1069 | Tagged RT-primer | GGC AGT GCG TGA ATA TGT TGC GGT GTG GGA CGC TAT GAG TT | 1267 |
|  | Gene-specific PCR-primer | GCC TCA CAT TCT CGA AGC TC |  |
|  | Tag PCR-primer | GGC AGT GCG TGA ATA TGT TGC |  |
|  |  |  |  |
| A4, PG0559 | Tagged RT-primer | GTC TAG CTC TCT CTA ATC GCC GGA TGC TAC AGC AGT GAA A | 234 |
|  | Gene-specific PCR-primer | GCA TCA GGA TCT TCC CAT CT |  |
|  | Tag PCR-primer | GTC TAG CTC TCT CTA ATC GC |  |
|  |  |  |  |
| A5, PG0775 | Tagged RT-primer | GCC TAG CTC TCT CTA ATT GCC GAA GCG AAG AAG GTA CCA C | 169 |
|  | Gene-specific PCR-primer | TAC GGT CTC CGC AAA GTT CT |  |
|  | Tag PCR-primer | GCC TAG CTC TCT CTA ATT GC |  |
|  |  |  |  |
| A5L, PG0775 | Tagged RT-primer | GGC AGT GCG TGA ATA TGT TGC GTA CGG GAA GGC GTA ATG AA | 1361 |
|  | Gene-specific PCR-primer | TAC GGT CTC CGC AAA GTT CT |  |
|  | Tag PCR-primer | GGC AGT GCG TGA ATA TGT TGC |  |
|  |  |  |  |
| *mutB,* PG1657 | RT-PCR F primer | ACA AGG CGT GGA CAC TCA TCA AG | 562 |
|  | RT-PCR R primer | TGC CCG TCC TGC CCC ATT TT |  |
|  |  |  |  |
| C1, PG1159 | Tagged RT-primer | GCC TTG CTT TCA CAG TTT GCG TCG ATT TTG GCA GCT ATC C | 146 |
|  | Gene-specific PCR-primer | CGA TGG AGT TGT AGC CCC TA |  |
|  | Tag PCR-primer | GCC TTG CTT TCA CAG TTT GC |  |
|  |  |  |  |
| C2, PG1144 | Tagged RT-primer | GCC TTG CTT TCA CAG TTT GCC ATC CAT AAC GGC ATT GAC A | 102 |
|  | Gene-specific PCR-primer | GAT AGA GTG GGG ATC GCA GA |  |
|  | Tag PCR-primer | GCC TTG CTT TCA CAG TTT GC |  |
|  |  |  |  |
| C3, novel^3^ | Tagged RT-primer | GGC AGT GCG TGA ATA TGT TGC ATC TGA CCA CAG GCA GGA AG | 252 |
|  | Gene-specific PCR-primer | GCG TGA TGA AGC GGT ATG TA |  |
|  | Tag PCR-primer | GGC AGT GCG TGA ATA TGT TGC |  |
|  |  |  |  |

**Table S1.** **Primer sequences.**

^1^ Primers were designed using Primer3 (http://frodo.wi.mit.edu/primer3) and OligoCalc: Oligonucleotide Properties Calculator (<http://www.basic.northwestern.edu/biotools/oligocalc.html>).

^2^ Tag primers were modified based on the sequence used by Purcell *et al.* .

^3^ Novel transcript found in an intergenic region between positions 2,226,192 to 2,226,442 nt, reverse strand.


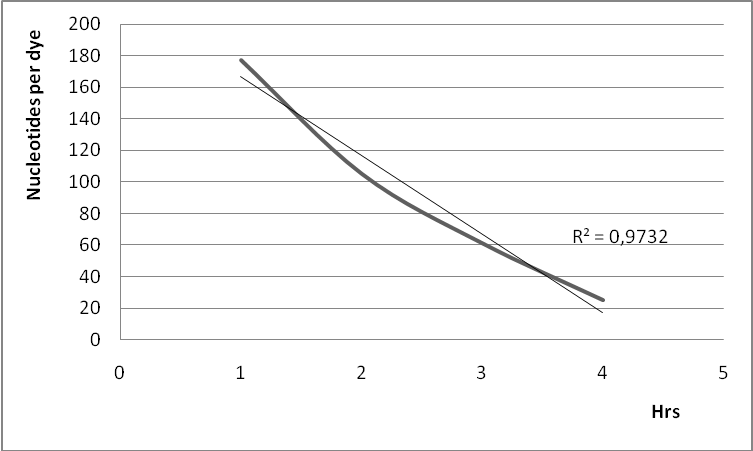


**Figure S1. Time course of RNA labeling by alkylation.** A time-series experiment of the chemical labeling was performed from 1 to 4 hours separately. The fluorescent densities of the four reactions were plotted and the linear correlation coefficient calculated. The fluorescent densities were represented by nucleotides per fluorescent dye.


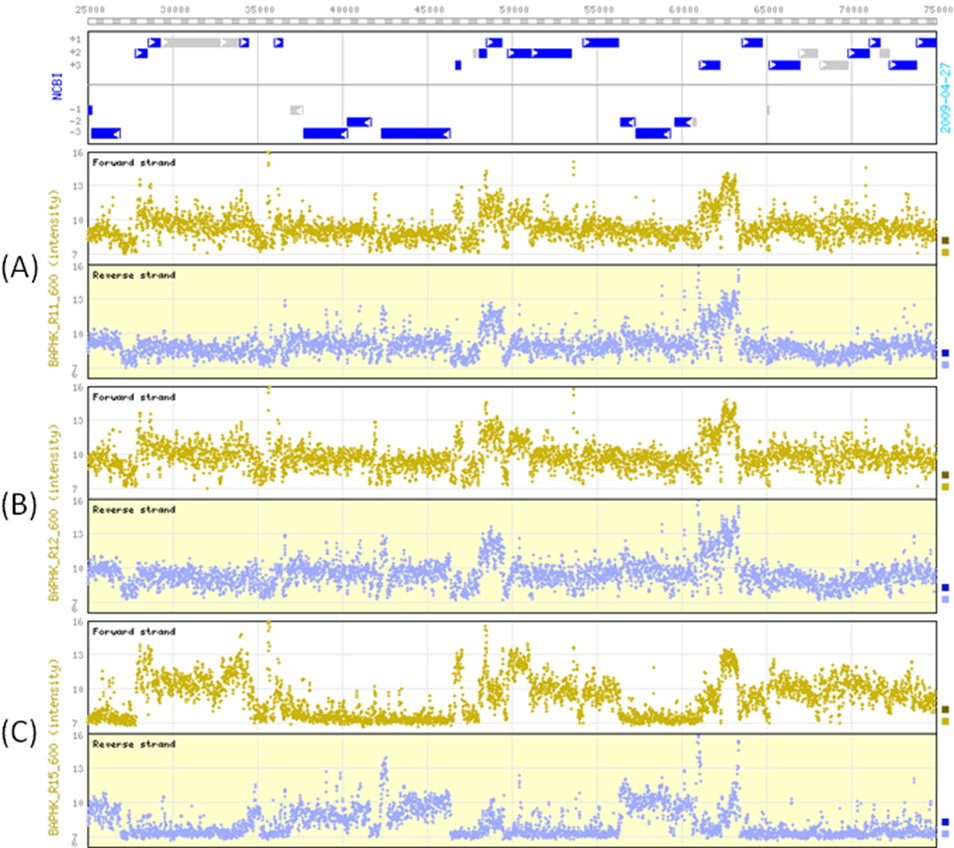


**Figure S2. The transcriptome profiles detected with different concentrations of salmon sperm DNA as blocking reagent.** Log2 probe intensities from a 50-kbp region of the *P. gingivalis* W83 genome were plotted. Top panel is the NCBI annotation of the genes in the range. Panels A, B and C were the transcriptome profiles detected with different concentrations of salmon sperm DNA at 0, 0.1, and 0.7 mg/ml respectively. Signals from the forward and reverse-complement strands were plotted in the upper and lower part of each panel respectively.

**
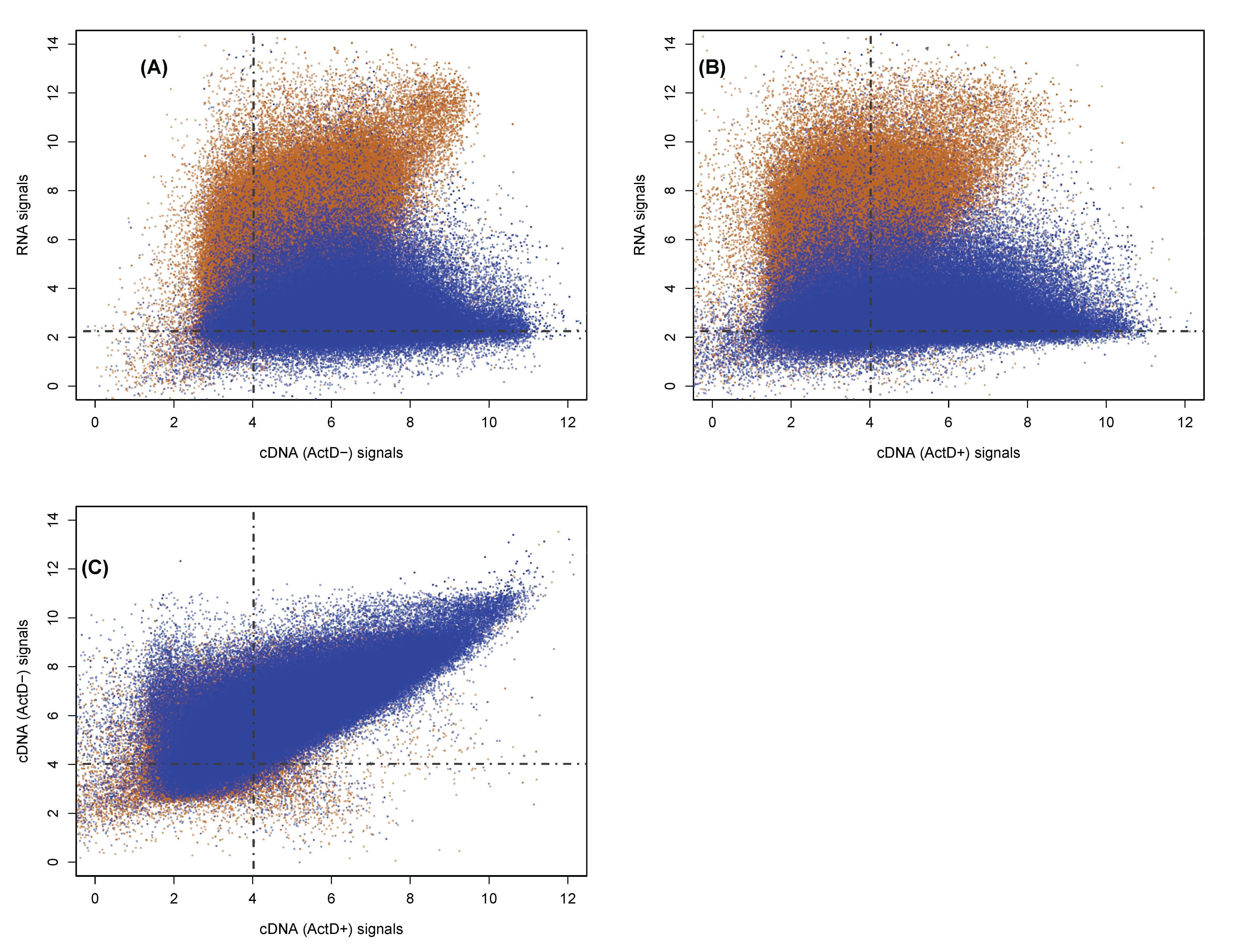
**

A

C

B

**Figure S3.** **Effects of actinomycin D on cDNA-based transcription signals.** Scatter plots

of the probe intensities between the RNA-based and the cDNA-based data without (A) and with (B) the addition of 6 µg/ml actinomycin D to the RT-reaction. Panel C is the scatter plot between the cDNA-based data generated with or without actinomycin D. Orange dots are probe signals from the ORF coding regions and blue the antisense regions. The dashed lines correspond to the baseline level of each intensity profile. The cDNA-based transcriptome profile is available at The Forsyth Institute’s “Microbial Transcriptome Database”, (<http://bioinformatics.forsyth.org/mtd>).

**
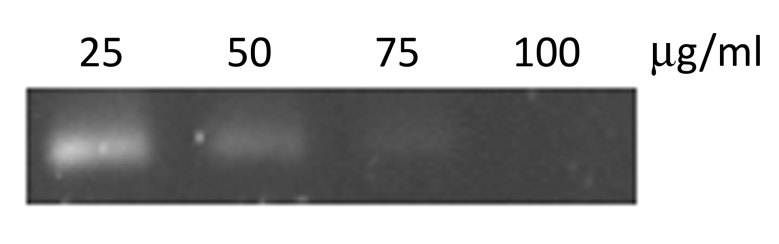
**

**Figure S4.** **Effects of actinomycin D on first-strand cDNA generation.** Increasing amount of actinomycin D was added to strand-specific RT-PCR for a highly expressed region (see Table S1, C3) and the results resolved by agarose gel electrophoresis. Concentrations of actinomycin D used in the reverse transcription are indicated.

**References**

1. Koehler A, Karch H, Beikler T, Flemmig TF, Suerbaum S, Schmidt H: **Multilocus sequence analysis of Porphyromonas gingivalis indicates frequent recombination**. *Microbiology* 2003, **149**(Pt 9):2407-2415.

2. Purcell MK, Hart SA, Kurath G, Winton JR: **Strand-specific, real-time RT-PCR assays for quantification of genomic and positive-sense RNAs of the fish rhabdovirus, Infectious hematopoietic necrosis virus**. *J Virol Methods* 2006, **132**(1-2):18-24.
